# Immunomodulation of the Natural Killer Cell Phenotype and Response during HCV Infection

**DOI:** 10.3390/jcm9041030

**Published:** 2020-04-06

**Authors:** Gaitan Fabrice Njiomegnie, Scott A. Read, Nicole Fewings, Jacob George, Fiona McKay, Golo Ahlenstiel

**Affiliations:** 1 Blacktown Clinical School and Research Centre, Western Sydney University, Blacktown 2148, NSW, Australias.read@westernsydney.edu.au (S.A.R.);; 2 Storr Liver Centre, Westmead Institute for Medical Research, University of Sydney, Westmead 2145, NSW, Australia; 3 Blacktown Hospital, Blacktown 2148, NSW, Australia; 4 Centre for Immunology and Allergy Research, Westmead Institute for Medical Research, Westmead 2145, NSW, Australia; 5 Westmead Clinical School, University of Sydney, Westmead 2145, NSW, Australia; 6 Westmead Hospital, Westmead 2145, NSW, Australia

**Keywords:** natural killer cell, hepatitis C virus, chronic infection, interferon, direct-acting antiviral

## Abstract

Hepatitis C virus (HCV) infection develops into chronic hepatitis in over two-thirds of acute infections. While current treatments with direct-acting antivirals (DAAs) achieve HCV eradication in >95% of cases, no vaccine is available and re-infection can readily occur. Natural killer (NK) cells represent a key cellular component of the innate immune system, participating in early defence against infectious diseases, viruses, and cancers. When acute infection becomes chronic, however, NK cell function is altered. This has been well studied in the context of HCV, where changes in frequency and distribution of NK cell populations have been reported. While activating receptors are downregulated on NK cells in both acute and chronic infection, NK cell inhibiting receptors are upregulated in chronic HCV infection, leading to altered NK cell responsiveness. Furthermore, chronic activation of NK cells following HCV infection contributes to liver inflammation and disease progression through enhanced cytotoxicity. Consequently, the NK immune response is a double-edged sword that is a significant component of the innate immune antiviral response, but persistent activation can drive tissue damage during chronic infection. This review will summarise the role of NK cells in HCV infection, and the changes that occur during HCV therapy.

## 1. Introduction

Hepatitis C virus (HCV) is a hepatotropic flavivirus and a major cause of chronic viral hepatitis, liver cirrhosis, and hepatocellular carcinoma (HCC) [1]. Approximately 55–85% of cases presenting with acute HCV infection progress to chronicity, with 20–30% developing liver cirrhosis, and 1–4% progressing to hepatocellular carcinoma [2]. Due to undiagnosed disease and lack of treatment availability, an estimated 1–3% of the world population currently lives with HCV infection [3]. The prevalence of HCV infection is particularly high in low-income countries within Asia (0.4–6.8%), North Africa/Middle East (2.5–3.9%), and sub-Saharan Africa (1.0–5.3%) [4,5]. Lower incidence is seen in the Oceania (0.1–1.5%), the Caribbean (0.2–1.3%), Europe (0.9–3.3%), and the Americas (0.9–1.9%) [4,5]. Despite these established ranges, countries such as Cameroon (13.8%), Burundi (11.3%), and Gabon (9.2%) have particularly high HCV seroprevalence due to limited availability of HCV screening and treatment strategies [4,5].

The antiviral immune response to hepatitis C is traditionally understood to be driven by the adaptive immune cells such as B cells and T cells, and more recently by innate immune cells, specifically natural killer (NK) cells. While adaptive immunity and hence antigenic specificity of B and T cells to viral infection is generated through somatic rearrangement of antigenic receptors, it is significantly slower than the innate immune response. NK cells possess an innate ability to provide a fast acting and potent response to cells deemed harmful, such as cancer and virus-infected cells, which is controlled by a number of NK cell receptors (discussed in detail below). This review will focus on the role NK cells play in HCV infection, inflammation, and fibrosis, as well as their potential use as biomarkers for antiviral response and future treatment strategies.

## 2. Natural Killer Cells

NK cells are lymphocytes that are derived from the bone marrow, develop in lymphoid tissue, and migrate into the bloodstream and tissue. Consequently, NK cells are present throughout the body and participate in the early defence against pathogens and cancer [6]. These innate lymphocytes can mount a rapid immune response (within hours to days) in the absence of specific antigen recognition [6]. This generally occurs before the induction of the adaptive immune response and is essential to control the spread of acute viral infections.

When compared to T and B cells, NK cells do not bear antigen-specific cell surface receptors in the traditional sense. For this reason, their function is not dependent on antigen presentation and/or clonal expansion. Instead, NK cells express a plethora of unique cell surface inhibitory and activating receptors (Table 1) [7]. High-dimensional analysis has revealed between 6000 and 30,000 combinatorial NK cell phenotypes can exist within an individual, with expression of inhibitory receptors largely genetically determined, and that of activating receptors primarily environmentally driven [8]. These receptors interact with ligands present on healthy or diseased cells to enact inhibitory or stimulatory NK cell activity, respectively. The cumulative signal mediated by these determines the NK cell response. If activated, NK cell effector functions are broadly divided into two categories: cytotoxic and cytokine-secreting. Cytotoxicity is mediated by the granule exocytosis pathway [6] or via death receptor pathways. The granule exocytosis pathway relies on delivery of serine proteases, called granzymes, into the target cell cytoplasm through pores formed by proteins perforin and granulysin. During degranulation, human NK cells release several of the five granzyme proteins (A, B, H, K, M; with varying protease specificities) which can induce caspase activation, mitochondrial dysfunction, or caspase-independent apoptosis (Reviewed in [9]).

NK cells can also kill target cells via engagement of death receptors on target cells. There are 3 different receptor/ligand systems: tumour necrosis factor-alpha (TNF-α), which binds TNF receptor-1 or -2, Fas ligand (FasL/CD95L) binding to CD95 (APO-1/Fas receptor) [39] and tumour necrosis factor-related apoptosis-inducing ligand (TRAIL), which engages various TRAIL receptors [40]. All three pathways may be used by NK cells, resulting in target cell apoptosis [9].

Importantly, NK cells can kill multiple targets in a serial fashion, using granule exocytosis for initial killing events, and the death-receptor pathway for later events when perforin and granzyme reserves are exhausted [41]. Further, target cytotoxicity can be mediated via antibody-dependent cellular cytotoxicity (ADCC) through the FcγRIII receptor CD16 [42]. In addition, secretion of immunomodulatory cytokines such as interferon-gamma (IFN-γ), and TNF-α also act to enhance NK cytotoxicity [43].

Nucleated cells express ligands for inhibitory receptors, such as class I major histocompatibility complex (MHC) molecules, on the cell surface. Consequently, NK cell activation is inhibited by interactions between MHC molecules on healthy cells and NK cell inhibitory killer cell immunoglobulin-like receptors (KIRs) [44]. Inhibitory receptor signalling is primarily mediated by immunoreceptor tyrosine-based inhibitory motifs (ITIM) located in the cytoplasmic tails of receptors. Indeed, the inhibitory interactions between KIR family members and NK cell inhibitory receptors natural killer group 2, member A (NKG2A) with class I human leukocyte antigen (HLA) molecules are mediated by ITIMs [45,46]. This interaction recruits Src homology-containing tyrosine phosphatase 1 (SHP-1), SHP-2, and SH2 domain-containing inositol-5-phosphatase (SHIP) enzymes that act to dephosphorylate signalling proteins Lck, Fyn, Syk, Zap70, and Vav1 to deactivate NK cells [47].

To evade antigenic recognition by the adaptive immune system, virus-infected and cancer cells downregulate class I MHC molecule expression [48]. However, loss of this ligand for its inhibitory receptors is the means by which the NK cell detects altered or “missing self”, thereby losing inhibitory input which can be permissive for NK cell activation. In addition, viral infection can stimulate the expression of activating receptors (see Table 1), resulting in a cumulative activation signal when recognized by NK cells. It is important to note that the activation of NK cells does not solely depend on the lack of MHC class I molecules. For NK cells to become fully activated, stress-induced activation receptors are vital. Most activating receptors signal via the immunoreceptor tyrosine-based activation motif (ITAM), which becomes phosphorylated by the Src family of tyrosine kinases (Lck, Fyn, Src, Yes, Fgr, and Lyn) upon ligand binding, resulting in the activation of tyrosine kinases Syk and Zap70 [7]. This activation generates a cascade of events stimulating the release of cytolytic granules containing perforin and granzymes and/or the production of immunostimulatory cytokines and chemokines [49].

In humans, NK cells are identified based on their expression of activating Fc receptor, CD16 (FcγRIIIA), CD56 and lack of CD3. Recent studies have classified the various stages of human NK cell development into six phases, of which the final three represent mature NK cells [49]. Phase 4 marks the transition of immature NKs into mature NK cells expressing NKp80, NKG2D, CD335, CD337, and CD161 [49]. Phase 5 represents circulating NK cells, and is characterised by an observable spike in CD56 expression representing the relatively young CD56^bright^ population, as well as the more mature CD56^dim^ population that co-express CD16 and killer immunoglobulin-like receptor (KIR) (CD158). Phase 6 represents terminal maturation gaining expression of CD57 and may include the generation of “adaptive” or “memory-like” NK cells following antigen exposure, gaining expression of NKG2C and KLRG1 [42,50].

Almost 10% of NK cells in the peripheral blood and roughly 100% of NK cells in secondary lymphoid tissues have a high surface expression of CD56 (CD56^bright^) and are CD16low, acting as potent immunoregulatory cells via the secretion of cytokines such as IFN-γ which contribute to the priming of T-helper cells type 1 (Th1) [51,52]. In contrast, approximately 90% of NK cells in the peripheral blood have a low surface expression of CD56 (CD56^dim^). CD56^dim^ NK cells express moderate-to-high levels of CD16 (CD16^bright^) and perforin, possessing high cytotoxic ability [6]. In addition to the above classification, a third subset of NK cells, CD56-CD16+, has been characterized [53]. This subset is rare and has been primarily observed in studies involving innate immunity against HIV infections [54], but also correlates with response to therapy in HCV infection [55].

The compartmentalization of NK cells throughout the body is likely due to the expression pattern of homing receptors. For example, CD56^bright^ NK cells display homing markers such as CCR7, CXCR6, CCR5, and CD62L for secondary lymphoid organs, while CD56^dim^ NK cells express homing markers that guide them towards inflamed or infected peripheral sites [51,56]. Peripheral organs such as the liver generally harbour more NK cells (up to 50% of resident lymphocytes), as opposed to 5–15% of NK cells in peripheral blood [57]. Liver-resident NK cells are Eomes^hi^ and are long-lived within the liver, being retained by hepatic expression of CCR5 ligands CCL3 and CCL5, and CXCR6 ligand CXCL16 [58,59]. Compared with circulating NK cells, liver-resident NK cells possess higher amounts of cytotoxic granzymes as well as apoptotic ligands such as TRAIL and FasL, supporting their apoptotic potential. Nonetheless, elevated hepatic IL-10 production makes the liver an immune-tolerant location, increasing the threshold of cytotoxic activation of resident cells.

Moreover, in response to HCV infection, NK cell concentration in the liver increases [60]. The infiltration of NK cells into the liver is mediated by chemokines such as CCL2, CXCL2, CXCL9, CXCL10, and a plethora of cytokines such as the TGF-β, IL-12, IL-18, secreted by hepatocytes, Kupffer cells, liver sinusoidal endothelial cells, and T cells [61,62]. As significant antiviral effectors in the liver, both resident and liver-infiltrating NK cells are a major component of the immune response against HCV infection, as outlined below. Nonetheless, the vast majority of studies examine circulating NK cells due to ease of sample collection, and therefore NK cells examined in this review will represent blood NK cells unless otherwise stated.

## 3. Interactions between NK Cells and HCV

The establishment of an in vitro HCV cell culture system employing the JFH-1 strain of virus (isolated from a Japanese man with fulminant hepatitis) has significantly advanced our understanding of NK cells as antiviral effectors, as well as the methods employed by HCV to circumvent the NK cell response [63]. In vitro co-culture studies using HCV-infected human hepatoma Huh-7.5 cells suggest that NK cell DNAM-1 is required for recognition of virus-infected cells, IFN-γ secretion, and cytolytic activity [64]. Consequently, NK cells have been shown to inhibit HCV replication in vitro directly via IFN-γ production, and via IFN-γ-mediated production of type I IFNs in Huh-7 cells [43]. Conversely, Huh-7 cells infected with the JFH-1 HCV strain have been shown to inhibit NK cell degranulation and IFN-γ production via downregulation of activating receptors NKG2D and NKp30 [65,66]. Indeed, NS5A has been shown to reduce NKG2D expression via increased monocyte production of TGFβ and reduced IL-12 [67]. Furthermore, co-culture with virus-infected Huh-7 cells was shown to downregulate the expression of both NKp30 and NKp46 and subsequent cytotoxic activity via the viral NS3 protease [65,68]. In vitro results have been corroborated in vivo, demonstrating a loss of NKG2D expression on NK cells in chronic hepatitis C (CHC) patients, as well as dampened cytotoxic and IFN-γ responses [69]. Binding of the HCV glycoprotein E2 to CD81 on NK cells has also been shown to suppress NK cell IFN-γ production [70]. In addition, HCV core protein increases the surface expression of HLA alpha chain E (HLA-E) on infected hepatocytes, reducing NK cell cytotoxicity via its interaction with the inhibitory receptor NKG2A [71,72].

**Figure 1:**
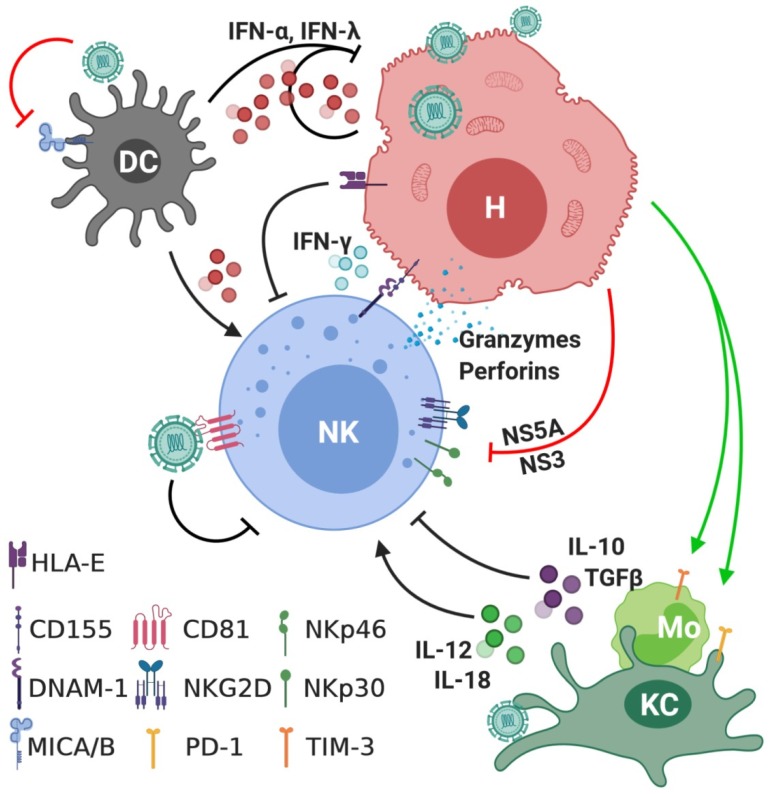
Hepatitis C virus (HCV)-mediated inhibition of NK function. In response to HCV infection, antiviral, immunomodulatory, and inflammatory cytokines such as IFN-α, IFN-λ, IL-12, and IL-18 are expressed by dendritic cells (DCs), hepatocytes (H), and myeloid populations both in circulation (monocytes, Mo) and within the liver (Kupffer cells, KC). Upon recognition of HCV-infected hepatocytes via DNAM-1, NK cells produce IFN-γ and release their cytotoxic granules to inhibit viral replication and kill infected cells, respectively. To minimise this NK response, viral proteins NS5A and NS3 reduce surface expression of activating receptors NKG2D, NKp30, and NKp46, whereas NKG2D ligands MICA/B are downregulated on DC populations. Moreover, core protein upregulated hepatocyte HLA-E that interacts with inhibitory NK receptor NKG2A and E2 interaction with CD81 inhibits NK cell IFN-γ production and cytotoxicity. Lastly, NK cell activating IL-12 and IL-18 secretion from monocytes and macrophage populations is reduced due to NS5A-mediated IL-10 production as well as HCV-mediated upregulation of PD-1 and Tim-3. Green line, upregulated expression; red line, downregulated expression.

Many of the interactions between HCV-infected hepatocytes and NK cells are regulated by cytokines secreted by monocytes, macrophages, conventional dendritic cells (cDCs), and plasmacytoid dendritic cells (pDCs) [60]. During HCV infection, Kupffer cells and pDCs play an essential role in sensing HCV RNA and activating NK cells via the secretion of innate cytokines (Figure 1) [73]. Notably, the interaction between pDCs and HCV-infected cells leads to type I and III IFN secretion [74]. While type I IFNs have been shown to have direct effects on HCV replication and NK cell activation, type I IFN mediated production of MICA/B expression on DCs was shown to be drastically reduced in HCV patients [75]. As a ligand for the activating receptor NKG2D, reduced MICA/B expression on DCs stimulated a subsequent reduction in NKG2D-mediated NK cell activation [75].

Acute HCV infection also stimulates monocyte/macrophage secretion of IL-12 and IL-18, as well as IL-15 from DC populations to stimulate NK cell activation [76]. However, inhibition of monocyte IL-12 secretion in CHC can result in upregulation/activation of PD-1- [77] or Tim-3-based [78,79] mechanisms, thus limiting NK cell activation. Alternatively, the viral NS5A protein has been shown to stimulate monocyte IL-10 secretion via toll-like receptor (TLR)-4. IL-10 inhibits the secretion of IL-12 and stimulates transforming growth factor (TGF)-β secretion, resulting in downregulation of NKG2D expression and inhibition of NK cell activity (Figure 1) [69]. Together, these data demonstrate that NK cell activity is modulated both directly through specific virus–host interactions, and indirectly by modulating the innate inflammatory and immune responses (Figure 1).

## 4. The Role of NK Cells in Acute Hepatitis C Infection

Early interaction between NK cell KIRs and host HLAs has been shown to have a significant impact on HCV clearance. KIRs vary in copy number and haplotype between individuals, and KIRs and their HLA cognate ligands reside on different chromosomes, such that certain KIR–HLA haplotype combinations exist in only a subset of the population. Combined inheritance of KIR2DL3 and HLA-C1 is associated with increased likelihood of viral clearance [80,81], possibly due to a lower avidity, reduced inhibitory NK signalling, and subsequently, more rapid NK cell activation [82]. In addition, the combination of KIR2DL3 and KIR2DS3 with HLA-C2 is associated with the development of chronic infection, the mechanism, however, remains unknown [83,84]. Lastly, individuals carrying the HLA-E^R^ allele (arginine) are more likely to clear HCV infection, possibly due to a lower affinity towards CD94–NKG2A than the HLA-E^g^ allele (glycine substitution) [72,84,85].

**Figure 2:**
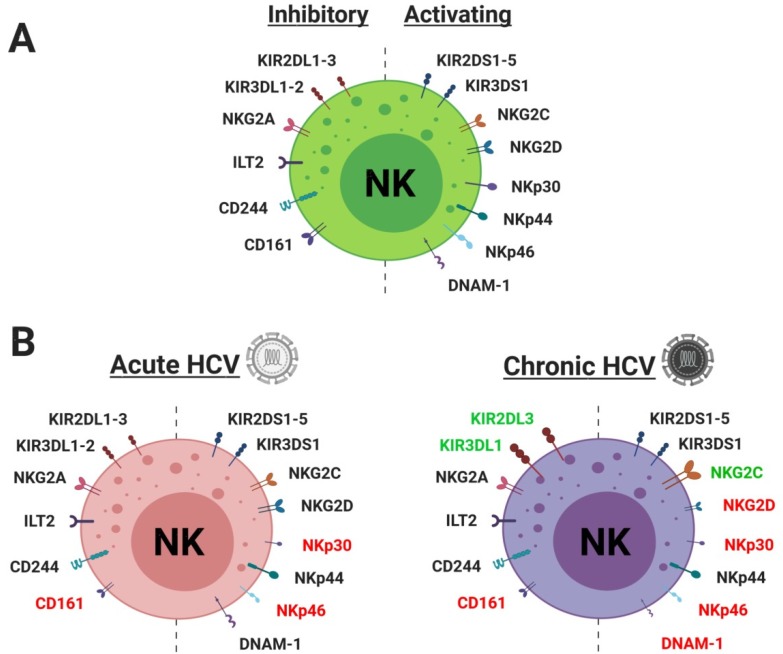
Modulation of NK cell inhibitory and activating receptor distribution following HCV infection. (**A**) NK cell inhibitory and activating receptor expression. Genetic (killer immunoglobulin-like receptors, KIRs) and environmental determinants drive expression of NK receptors, resulting in significant diversity within the circulating NK cell populations. (**B**) Modulation of NK cell receptor distribution following acute and chronic HCV infection. Red text denotes a reduction in circulating NK cells expressing a given receptor, and green text denotes an increase in these. Unfortunately, due to a lack of evidence, it is not known whether these changes are due to NK infiltration into the liver (and hence reduction in the blood), infection-mediated alterations in receptor expression, or both.

Acute HCV infection results in a reduction in circulating CD56^dim^ NK cells and expansion of CD56^bright^ NK cells [86]. It remains unknown whether this represents differential distribution (i.e., trafficking) of the subsets, or a systemically altered phenotype. The frequency of NK cells expressing activation receptors NKp46 and NKp30 is significantly decreased in acute infection compared with healthy controls [87] (Figure 2). Spontaneous clearance also appears to be associated with a decreased frequency of NKp46 and NKp30, as well as NKG2D and CD161 in acute HCV infection [87]. Moreover, an increased frequency of NKG2A and NKG2D NK cells in addition to decreased NKG2C and CD158e frequency in HCV–HIV co-infection is associated with HCV clearance [88]. Together, these data strongly suggest that multiple activating receptors likely contribute to viral clearance via migration into the liver, hence their relative reduction in the blood. This is unlikely to be confirmed unfortunately, as intrahepatic NK cells in acute HCV infection must be collected, and biopsy at this time point is unlikely.

## 5. The Role of NK Cells in Chronic Hepatitis C (CHC) Infection

Once CHC is established, a significant alteration in NK cell phenotype, distribution, and composition is observed. Similar to acute infection, the frequency of circulating CD56^dim^ CD16+ and CD56^bright^ NK cells becomes decreased and increased, respectively [87,89]. Moreover, the proportion of CD56-CD16+ NK cells with defective function becomes elevated [87,90]. In contrast to acute HCV infection, NK cells remain continuously activated in CHC, however this activation does not consistently increase all NK cell functionalities [91]. Cytotoxicity becomes enhanced in CHC, but the capacity to produce IFN-γ and TNFα is impaired [90,92]. This phenotypic change is likely the result of chronic endogenous IFN-α exposure, resulting in the preferential phosphorylation of STAT1 over STAT4 [93,94,95]. This change in STAT phosphorylation results in an increase in STAT1-mediated cytotoxicity and impaired IFN-γ production, which is mediated via STAT4 activation. In line with these data, there is a reduction in perforin+ CD56^dim^ NK cells in CHC, supporting excessive degranulation during chronic infection [90]. Chronic liver damage in CHC has been associated with the NKp46^high^ subset of NK cells that accumulate in the liver in CHC and correlate with inflammation [96]. Nonetheless, this population has also been shown to mediate stellate cell apoptosis and is inversely associated with fibrosis stage, suggesting that NKp46^high^ NK cells may possess antifibrotic activities as well [97].

NK cell activation and inhibitory receptor expression also becomes significantly altered in CHC patients. The frequency of NK cells expressing inhibitory receptor CD94/NKG2A and activating receptor NKG2C increases in CHC [98,99], whereas the frequency of natural cytotoxicity receptor (NCR) NKp30 and NKp46 as well as DNAM-1-expressing NK cells is reduced [87,100]. NKG2C+ cell expansion has been linked to co-infection with human cytomegalovirus (HCMV) and chronic hepatitis B virus (HBV) or HCV, suggesting that it may not be an HCV-specific mechanism [101]. In addition, NKG2A has recently been shown to stimulate NK cell exhaustion via its interaction with HCV-upregulated Qa-1 in mice [102]. Treatment with anti-NKG2A or anti-Qa-1 monoclonal antibodies was shown to enhance NK cell-dependent HCV clearance via increased cytotoxicity and secretion of IFN-γ, resulting in stimulation of the T cell response [102]. NKG2D+ NK cells also reduce in frequency and return to normal upon successful treatment [87,90]. While genetic studies dominate the examination of KIR biology, surface expression of inhibitory receptor KIR2DL3-expressing NK cells has been shown to increase in CHC, and KIR3DL1-expressing cells decrease [99]. Moreover, KIR2DL1, 2DL2, and 2DS1 show no change in frequency in CHC [103]. KIR3DS1 surface expression has not been examined with respect to HCV infection, however, carriers of KIR3DS1 are more likely to achieve sustained virologic response (SVR; defined here as undetectable serum HCV RNA at 24 weeks after completion of antiviral therapy) [104] and are less likely to develop HCC [105]. Recently, the KIR3DS1–HLA-F interaction was shown to significantly reduce viral replication in vitro, supporting its antiviral function [106].

Understanding the NK phenotype during HCV infection has been overshadowed by the impressive clearance rates following the implementation of direct-acting antiviral (DAA) treatments for chronic infection. Nonetheless, it is vital to understand changes in NK function in response to chronic viral infections. This will allow therapeutic interventions aimed at strengthening the NK response to current problem pathogens, including HBV. For example, a mature NK phenotype expressing CD57, KIRs and reduced NKG2A has been associated with spontaneous HCV clearance [107]. Interestingly, blocking NKG2A expression in murine hepatic NK cells enables a stronger antiviral CD8+ T cell response by improving NK cell IFN-γ production and DC antigen presentation [108]. Harnessing the antiviral nature of terminally differentiated NK cells may facilitate clearance of chronic viral infections as part of the classical innate immune response as well as in a pathogen-specific manner. Pathogen-specific NK responses have recently been shown in a number of animal models [109], warranting further examination of the mechanisms by which NK memory is generated, and the receptors responsible for pathogen interactions.

## 6. NK Cell Memory in HCV

For some time, immune memory has been considered a unique feature of B and T lymphocytes, enabling them to provide long-term protection against previously encountered pathogens. Classically, NK cells have been excluded from the adaptive immune response due to their inability to generate antigen-specific receptors following somatic rearrangement. Their collection of activating and inhibitory receptors has long been considered a significant driver of the innate immune response; however recent studies have demonstrated their potential for driving immunological memory. Specifically, mouse, primate, and human studies have demonstrated an antigen-specific and long-lived recall response against such viral antigens as human cytomegalovirus (HCMV), simian immunodeficiency virus (SIV), and Hantavirus [110,111,112]. These responses are mediated primarily by an NK population expressing KLRG1, utilizing specific NK cell receptors such as NKG2C and NKG2A that are indispensable for memory responses against CMV and SIV, respectively [111,112,113].

A number of studies have identified the liver as a key reservoir for memory NK cells [114,115], and yet they have been overlooked in the context of hepatic viral infections. While there are no published studies examining the memory response to HBV or HCV, our group has identified an expanded population of KLRG1+ NK cells in chronic HBV (CHB) patients that possess antifibrotic activity [42]. We have since further characterized this population and have identified a memory population of NK cells in CHB patients that degranulate in response to HBV antigen [116]. Importantly, strength of degranulation was inversely associated with viral titre, suggesting that memory NK cells in this context possess an antiviral role. We have also identified a population of KLRG1+ NK cells that develop in response to chronic HCV infection (manuscript under review): in response to HCV antigens (JFH1 infected Huh-7 cells) these memory NK cells undergo proliferation as well as degranulation that is absent in healthy controls. Together, these data indicate that HCV infection can stimulate NK cell memory, however their pathogenic relevance and therapeutic potential are unclear. A better understanding of the mechanisms by which NK cell memory is developed will help guide vaccine development to stimulate a potent HCV adaptive immune response.

## 7. The Effect of Anti-HCV Therapy on NK Cell Phenotype and Activity

Treatment of HCV infection aims to achieve viral clearance through sustained virologic response (SVR), i.e., reducing circulating HCV RNA to an undetectable level at least three months following the cessation of therapy. Traditionally, CHC infection has been treated with pegylated IFN-α (PEG-IFN-α) in combination with ribavirin, a nucleoside analogue, with an SVR rate of 50–60% [117]. Apart from the relatively poor response rate, IFN-ribavirin treatment was associated with a number of adverse effects, including anemia, flu-like symptoms, and depression. In 2011, the first two DAAs against the HCV protease, boceprevir and telaprevir, were approved [118,119], demonstrating a significantly favourable increase in SVR when compared with pegylated IFN-α/ribavirin-based therapy alone in individuals infected with HCV genotype 1. Current DAA-based therapies are IFN-free, pan-genotypic, and highly tolerable regimens, resulting in SVR in >95% of cases [120,121]. Nonetheless, IFN-based regimens have taught us much on the role of NKs in the antiviral immune response to HCV and will be examined below.

### 7.1. Effect of Pegylated IFN-α/Ribavirin-Based Therapy on NK Cells

IFN-α is a potent activator of antiviral immunity, of which NK cells are a critical component. Consequently, the expression of activating and inhibitory receptors, as well as the KIR and HLA haplotypes are significant contributing factors to treatment response. Surprisingly, increased baseline presence of activating receptor NKp30-, NKp46-, and NKG2D+ NK cells and decreased NKG2A is associated with non-response to IFN-based therapy [96,100,122,123]. This is in line with the IFN-refractory state of nonresponders, who demonstrate a pre-activated IFN response signature in the liver, and minimal response to exogenous IFN-α treatment [124]. In support of these findings, higher baseline expression of the IFN-α receptor, IFNAR1, is associated with improved response to treatment, in an IFNL3 genotype-dependant manner [125]. Importantly, while nonresponders do not upregulate NKp30 or NKp46 in response to IFN treatment, responders demonstrate an increase in NKp30 or NKp46 following 12 weeks of treatment [96]. Contrarily, the frequency of KIR2DL3+ NK cells is elevated in patients with early virological response, and significantly decreases only in SVR patients, not their nonresponsive counterparts [123]. This is supported by genetic studies demonstrating an association between KIR2DL3 with SVR and KIR2DL2 with non-response [126]. In addition to KIR variants, the *HLA-C* C2C2 genotype and *IFNL3* rs8099917 G allele demonstrated additive predictive value with regard to SVR in genotype 1 patients [127].

Lastly, responder NK cells also demonstrate an increased pretreatment expression of perforin compared with nonresponders that remains elevated for the first 12 weeks of treatment [128]. Rapid virological response was also found to be associated with an increase in CD69-expressing cells throughout the first 12 weeks of treatment [128].

The NK response to IFN treatment has also been examined early in treatment, demonstrating a fast and potent upregulation of activating receptor NKG2D as well as the NK activation marker CD69 within 24 h of IFN treatment initiation [40]. This correlates with an increase in NK degranulation and TRAIL expression at 24 h, driving the increase in blood alanine transaminase (ALT; a surrogate measure of liver inflammation or injury) that is likely the result of killing of infected hepatocytes. Increased NK TRAIL expression, and to a lesser degree, degranulation, was associated with phosphorylated STAT1 after 6 h post-IFN treatment initiation [95]. Conversely, an inverse association was noted for IFN-γ-producing cells, suggesting that IFN-α-induced STAT1 phosphorylation polarises NK cells towards a cytotoxic phenotype as compared with IFN-γ production. These data support the IFN-refractory phenotype of nonresponders, demonstrating that an increase in STAT1 phosphorylation in NK cells was associated with a decrease in viral titre [95].

In summary, these data suggest that NK cells play a key role in IFN-based antiviral treatments, particularly at the early stages. Activation and inhibitory receptor expression play a key role in NK cell activity, and their modulation by IFN-α is vital to stimulate NK cell antiviral activity.

### 7.2. Effect of Direct-Acting Antivirals-Based Therapy on NK Cells

The first data showing that NK cell function can be modulated by non-interferon-based antivirals was reported by Werner et al. in 2014, prior to the clinical use of direct-acting antivirals (DAAs) [129]. Ribavirin monotherapy improved NK cell STAT4 phosphorylation in vitro and in vivo and hence normalized NK cell IFN-γ secretion, suggesting that the previously reported HCV-related polarized NK cell phenotype is reversible [90,91].

The advent of DAAs for HCV has revolutionised the treatment of chronic of infection. IFN-free, DAA-based treatments show eradication rates of >95% regardless of viral genotype, replacing SVR rates of 50% common among IFN-based therapies [130]. Unfortunately, while SVR rates are high with DAAs, DAAs do not appear to induce neutralizing immunity and thus protection from re-infection. Nevertheless, important immune effects have been reported with IFN-free, DAA-based therapy: Martin et al. reported in 2014 that HCV eradication in the absence of IFN therapy results in enhanced frequency of HCV-specific cytotoxic CD8+ T cells in parallel with rapidly declining HCV RNA levels [131]. Subsequent to this, Serti et al. reported a rapid decrease in HCV viral load and level of inflammatory cytokines by week 8 in patients with SVR on asunaprevir + daclatasvir treatment [132]. This was accompanied by a reduction in activation levels of intrahepatic and blood NK cells and subsequent normalization of NK cell phenotype and function. Spaan et al. described in patients receiving the same DAA regimen a similar reduction in NK cell expression of activating NK cell receptors NKp30 and NKp46 as well the inhibiting receptor NKG2A [133]. In addition, they found a decrease in NK cell TRAIL expression, suggesting that the polarized, activated NK cell phenotype was rapidly normalizing with reduction in viral loads and related reduction in NK cell-activating cytokines such as IL-12 and IL-18 [133].

This was further evaluated by Golden-Mason et al., who observed a significant reduction of NK cell activation as measured by CD69 and a reduction of the less mature CD56^bright^ NK cell subset within 2 weeks of ledipasvir/sofosbuvir treatment, resulting in a sustained normalization of the NK cell phenotype [134]. Alao et al. complemented these results mechanistically by examining blood and liver specimen of patients at baseline and day 1 of treatment with asunaprevir + daclatasvir [135]: Patients with subsequent SVR as compared with treatment failure had higher hepatic baseline interferon-stimulated gene (ISG) expression and higher frequency of activated TRAIL-expressing and actively degranulating NK cell cells in blood at baseline and day 1 of treatment.

While a number of studies have described a dampening or normalization of NK cell phenotype and function in response to DAA therapy, there is still some question to what degree HCV introduces a long-lasting change in NK cell diversity. Strunz et al. employed high-dimensional flow cytometry combined with stochastic neighbour embedding analysis to show that HCV infection imprints on NK cells, a phenomenon that appears to persist after HCV eradication [136]. In another study, HCV-related microRNAs were found to be enriched in exosomes of patents with HCV infection and decreased markedly with DAA-based therapy. These microRNAs had immunomodulatory impact on NK cell function and, to some degree, on treatment and post-treatment changes in NK cell phenotype and function [137].

Finally, there has been significant concern about a possible negative impact of DAA-based therapy on HCC occurrence and recurrence. Notably, current expert opinion states that there is no conclusive evidence to support earlier concerns that DAA-based therapy influences risk, time to, or aggressiveness of HCC recurrence in patients that had successful HCC therapy. Chu et al. reported that higher pre-treatment levels of NKG2D on NK cells and rapid decline on treatment were one of several risk factors for early HCC after DAA-based therapy [138]. Importantly, other risk factors were higher fibrotic scores, previous HCC, and treatment failure, which are all well-recognized risk factors for HCC [138].

## 8. Conclusions

The innate immune system is an important player in antiviral immunity to HCV infection. Especially in the face of exhaustion/loss of potency of the T and B cell responses, NK cells are critical in protective immunity against HCV infection via modulation of their activating and inhibiting receptors. In chronic infection, HCV significantly influences the NK cell compartment, receptor repertoire, and effector functions. Consequently, alterations in the NK cell repertoire are noted in acute HCV infection and contribute to the progression of CHC. This inflammatory progression is related to a polarized NK cell phenotype with enhanced cytotoxicity but loss of effective IFN-γ secretion. This renders the NK cell inefficient at clearing virus but results in persistent liver damage and inflammation. Understanding the cellular and chemical mediators of this change, and the resulting effects on NK cells, will benefit the development of future vaccines as well as curative and preventative treatments for HCV. This will certainly include a better understanding on NK cell memory in HCV infection, which has the potential to revolutionize vaccine development in the near future.

## Figures and Tables

**Table 1:** Activating and inhibitory human natural killer (NK) cell receptors.

Types	Receptor	Ligand	Ref (Ligand)	Acute	Chronic	In Vitro	Ref (Expression)
**Activating**	NKG2D	MICA/B, ULBP1-6	[10,11]	↔	↓	↓	Yoon et al., 2011; Sene et al., 2010; Alter et al., 2011
**Receptor**	CD94-NKG2C	HLA-E	[12]		↑		Szereday et al., 2016

	KIR2DL4	HLA-G	[13]				

	KIR2DS1	HLA-C2	[14]		↔		Cosgrove et al., 2014

	KIR2DS2	HLA-A11	[15]				

	KIR2DS3	Unknown					

	KIR2DS4	HLA-A11, HLA-C05:01	[16,17]				

	KIR2DS5	Unknown					

	KIR3DS1	HLA-F	[18]				

	NKp30	B7H6, BAT3, HCMV pp65, HS	[19,20,21,22]	↓	↓	↓	Yoon et al., 2016; Holder et al., 2013; Alter et al., 2011

	NKp46	Heparin, viral HA and HN, CFP	[21,23,24,25]	↓	↓	↓	Sene et al., 2010; Alter et al., 2011

	NKp44	Viral HA and HN, PCNA, HS	[21,25,26,27]	↔	↔		Alter et al., 2011

	DNAM-1	CD112, CD155	[28]		↓	↓	Yoon et al., 2016; Bozzano et al., 2011

**Inhibiting**	KIR2DL1	HLA-C2	[14]	↔	↔		Alter et al., 2011; Cosgrove et al., 2014
**Receptor**	KIR2DL2	HLA-C1	[29]		↔		Cosgrove et al., 2014
	KIR2DL3	HLA-C1	[30]		↑		Szereday et al., 2016

	KIR2DL4	HS	[31]				

	KIR3DL1	HLA-Bw4	[32]		↓		Oliviero et al., 2009; Szereday et al., 2016

	KIR3DL2	HLA-A3-A11	[33]				

	CD94-NKG2A	HLA-E	[12]	↔	↔	↔	Holder et al., 2013; Alter et al., 2011

	ILT2 (CD85j)	MHC-I, HCMV UL18, S100A9	[34,35,36]		↔		Oliviero et al., 2009; Szereday et al., 2016

	CD244 (2B4)	CD48	[37]		↔	↔	Yoon et al., 2016; Cosgrove et al., 2014

	CD161 (KLRB1)	LLT1	[38]	↓	↓		Alter et al., 2011; Cosgrove et al., 2014


CFP, complement factor P; HA, hemagglutinin; HN, hemagglutinin neuraminidase; HS, heparan sulfate; PCNA, proliferating cell nuclear antigen; ↓ decreased frequency of receptor positive cells in blood, ↑ increased frequency of receptor positive cells in blood, ↔ no difference in blood.

## References

[1] Shepard C.W., Finelli L., Alter M.J. (2005). Global epidemiology of hepatitis C virus infection. Lancet Infect. Dis..

[2] Lingala S., Ghany M.G. (2015). Natural History of Hepatitis C. Gastroenterol. Clin. N. Am..

[3] Averhoff F.M., Glass N., Holtzman D. (2012). Global burden of hepatitis C: Considerations for healthcare providers in the United States. Clin. Infect. Dis..

[4] Gower E., Estes C., Blach S., Razavi-Shearer K., Razavi H. (2014). Global epidemiology and genotype distribution of the hepatitis C virus infection. J. Hepatol..

[5] Petruzziello A., Marigliano S., Loquercio G., Cozzolino A., Cacciapuoti C. (2016). Global epidemiology of hepatitis C virus infection: An up-date of the distribution and circulation of hepatitis C virus genotypes. World J. Gastroenterol..

[6] Mandal A., Viswanathan C. (2015). Natural killer cells: In health and disease. Hematol. Oncol. Stem. Cell Ther..

[7] Long E.O., Kim H.S., Liu D., Peterson M.E., Rajagopalan S. (2013). Controlling natural killer cell responses: Integration of signals for activation and inhibition. Annu. Rev. Immunol..

[8] Horowitz A., Strauss-Albee D.M., Leipold M., Kubo J., Nemat-Gorgani N., Dogan O.C., Dekker C.L., Mackey S., Maecker H., Swan G.E. (2013). Genetic and Environmental Determinants of Human NK Cell Diversity Revealed by Mass Cytometry. Sci. Transl. Med..

[9] Prager I., Watzl C. (2019). Mechanisms of natural killer cell-mediated cellular cytotoxicity. J. Leukoc. Biol..

[10] Cosman D., Mullberg J., Sutherland C.L., Chin W., Armitage R., Fanslow W., Kubin M., Chalupny N.J. (2001). ULBPs, novel MHC class I-related molecules, bind to CMV glycoprotein UL16 and stimulate NK cytotoxicity through the NKG2D receptor. Immunity.

[11] Bauer S., Groh V., Wu J., Steinle A., Phillips J.H., Lanier L.L., Spies T. (1999). Activation of NK cells and T cells by NKG2D, a receptor for stress-inducible MICA. Science.

[12] Braud V.M., Allan D.S., O’Callaghan C.A., Soderstrom K., D’Andrea A., Ogg G.S., Lazetic S., Young N.T., Bell J.I., Phillips J.H. (1998). HLA-E binds to natural killer cell receptors CD94/NKG2A, B and C. Nature.

[13] Rajagopalan S., Long E.O. (1999). A human histocompatibility leukocyte antigen (HLA)-G-specific receptor expressed on all natural killer cells. J. Exp. Med..

[14] Cognet C., Farnarier C., Gauthier L., Frassati C., Andre P., Magerus-Chatinet A., Anfossi N., Rieux-Laucat F., Vivier E., Schleinitz N. (2010). Expression of the HLA-C2-specific activating killer-cell Ig-like receptor KIR2DS1 on NK and T cells. Clin. Immunol..

[15] Liu J.X., Xiao Z.W., Ko H.L., Shen M.X., Ren E.C. (2014). Activating killer cell immunoglobulin-like receptor 2DS2 binds to HLA-A*11. Proc. Natl. Acad. Sci. USA.

[16] Sim M.J.W., Rajagopalan S., Altmann D.M., Boyton R.J., Sun P.D., Long E.O. (2019). Human NK cell receptor KIR2DS4 detects a conserved bacterial epitope presented by HLA-C. Proc. Natl. Acad. Sci. USA.

[17] Graef T., Moesta A.K., Norman P.J., Abi-Rached L., Vago L., Aguilar A.M.O., Gleimer M., Hammond J.A., Guethlein L.A., Bushnell D.A. (2009). KIR2DS4 is a product of gene conversion with KIR3DL2 that introduced specificity for HLA-A*11 while diminishing avidity for HLA-C. J. Exp. Med..

[18] Garcia-Beltran W.F., Holzemer A., Martrus G., Chung A.W., Pacheco Y., Simoneau C.R., Rucevic M., Lamothe-Molina P.A., Pertel T., Kim T.E. (2016). Open conformers of HLA-F are high-affinity ligands of the activating NK-cell receptor KIR3DS1. Nat. Immunol..

[19] Brandt C.S., Baratin M., Yi E.C., Kennedy J., Gao Z., Fox B., Haldeman B., Ostrander C.D., Kaifu T., Chabannon C. (2009). The B7 family member B7-H6 is a tumor cell ligand for the activating natural killer cell receptor NKp30 in humans. J. Exp. Med..

[20] Arnon T.I., Achdout H., Levi O., Markel G., Saleh N., Katz G., Gazit R., Gonen-Gross T., Hanna J., Nahari E. (2005). Inhibition of the NKp30 activating receptor by pp65 of human cytomegalovirus. Nat. Immunol..

[21] Hecht M.L., Rosental B., Horlacher T., Hershkovitz O., De Paz J.L., Noti C., Schauer S., Porgador A., Seeberger P.H. (2009). Natural Cytotoxicity Receptors NKp30, NKp44 and NKp46 Bind to Different Heparan Sulfate/Heparin Sequences. J. Proteome Res..

[22] Von Strandmann E.P., Simhadri V.R., von Tresckow B., Sasse S., Reiners K.S., Hansen H.P., Rothe A., Boll B., Simhadri V.L., Borchmann P. (2007). Human leukocyte antigen-B-associated transcript 3 is released from tumor cells and engages the NKp30 receptor on natural killer cells. Immunity.

[23] Mandelboim O., Lieberman N., Lev M., Paul L., Arnon T.I., Bushkin Y., Davis D.M., Strominger J.L., Yewdell J.W., Porgador A. (2001). Recognition of haemagglutinins on virus-infected cells by NKp46 activates lysis by human NK cells. Nature.

[24] Narni-Mancinelli E., Gauthier L., Baratin M., Guia S., Fenis A., Deghmane A.E., Rossi B., Fourquet P., Escaliere B., Kerdiles Y.M. (2017). Complement factor P is a ligand for the natural killer cell-activating receptor NKp46. Sci. Immunol..

[25] Jarahian M., Watzl C., Fournier P., Arnold A., Djandji D., Zahedi S., Cerwenka A., Paschen A., Schirrmacher V., Momburg F. (2009). Activation of Natural Killer Cells by Newcastle Disease Virus Hemagglutinin-Neuraminidase. J. Virol..

[26] Rosental B., Hadad U., Brusilovsky M., Campbell K.S., Porgador A. (2012). A novel mechanism for cancer cells to evade immune attack by NK cells The interaction between NKp44 and proliferating cell nuclear antigen. Oncoimmunology.

[27] Arnon T.I., Lev M., Katz G., Chernobrov Y., Porgador A., Mandelboim O. (2001). Recognition of viral hemagglutinins by NKp44 but not by NKp30. Eur. J. Immunol..

[28] Bottino C., Castriconi R., Pende D., Rivera P., Nanni M., Carnemolla B., Cantoni C., Grassi J., Marcenaro S., Reymond N. (2003). Identification of PVR (CD155) and nectin-2 (CD112) as cell surface ligands for the human DNAM-1 (CD226) activating molecule. J. Exp. Med..

[29] Snyder G.A., Brooks A.G., Sun P.D. (1999). Crystal structure of the HLA-Cw3 allotype-specific killer cell inhibitory receptor KIR2DL2. Proc. Natl. Acad. Sci. USA.

[30] Mandelboim O., Reyburn H.T., ValesGomez M., Pazmany L., Colonna M., Borsellino G., Strominger J.L. (1996). Protection from lysis by natural killer cells of group 1 and 2 specificity is mediated by residue 80 in human histocompatibility leukocyte antigen C alleles and also occurs with empty major histocompatibility complex molecules. J. Exp. Med..

[31] Brusilovsky M., Cordoba M., Rosental B., Hershkovitz O., Andrake M.D., Pecherskaya A., Einarson M.B., Zhou Y., Braiman A., Campbell K.S. (2013). Genome-Wide siRNA Screen Reveals a New Cellular Partner of NK Cell Receptor KIR2DL4: Heparan Sulfate Directly Modulates KIR2DL4-Mediated Responses. J. Immunol..

[32] Gumperz J.E., Litwin V., Phillips J.H., Lanier L.L., Parham P. (1995). The Bw4 Public Epitope of Hla-B Molecules Confers Reactivity with Natural-Killer-Cell Clones That Express Nkb1, a Putative Hla Receptor. J. Exp. Med..

[33] Hansasuta P., Dong T., Thananchai H., Weekes M., Willberg C., Aldemir H., Rowland-Jones S., Braud V.M. (2004). Recognition of HLA-A3 and HLA-A11 by KIR3DL2 is peptide-specific. Eur. J. Immunol..

[34] Arnold V., Cummings J.S., Moreno-Nieves U.Y., Didier C., Gilbert A., Barre-Sinoussi F., Scott-Algara D. (2013). S100A9 protein is a novel ligand for the CD85j receptor and its interaction is implicated in the control of HIV-1 replication by NK cells. Retrovirology.

[35] Chapman T.L., Heikema A.P., West A.P., Bjorkman P.J. (2000). Crystal structure and ligand binding properties of the D1D2 region of the inhibitory receptor LIR-1 (ILT2). Immunity.

[36] Colonna M., Navarro F., Bellon T., Llano M., Garcia P., Samaridis J., Angman L., Cella M., LopezBotet M. (1997). A common inhibitory receptor for major histocompatibility complex class I molecules on human lymphoid and myelomonocytic cells. J. Exp. Med..

[37] Latchman Y., McKay P.F., Reiser H. (1998). Cutting edge: Identification of the 2B4 molecule as a counter-receptor for CD48. J. Immunol..

[38] Aldemir H., Prod’homme V., Dumaurier M.J., Retiere C., Poupon G., Cazareth J., Bih F., Braud V.M. (2005). Cutting edge: Lectin-like transcript 1 is a ligand for the CD161 receptor. J. Immunol..

[39] Glassner A., Eisenhardt M., Kramer B., Korner C., Coenen M., Sauerbruch T., Spengler U., Nattermann J. (2012). NK cells from HCV-infected patients effectively induce apoptosis of activated primary human hepatic stellate cells in a TRAIL-, FasL- and NKG2D-dependent manner. Lab. Investig..

[40] Ahlenstiel G., Edlich B., Hogdal L.J., Rotman Y., Noureddin M., Feld J.J., Holz L.E., Titerence R.H., Liang T.J., Rehermann B. (2011). Early changes in natural killer cell function indicate virologic response to interferon therapy for hepatitis C. Gastroenterology.

[41] Prager I., Liesche C., van Ooijen H., Urlaub D., Verron Q., Sandstrom N., Fasbender F., Claus M., Eils R., Beaudouin J. (2019). NK cells switch from granzyme B to death receptor-mediated cytotoxicity during serial killing. J. Exp. Med..

[42] Wijaya R.S., Read S.A., Schibeci S., Eslam M., Azardaryany M.K., El-Khobar K., van der Poorten D., Lin R., Yuen L., Lam V. (2019). KLRG1+ natural killer cells exert a novel antifibrotic function in chronic hepatitis B. J. Hepatol..

[43] Wang S.H., Huang C.X., Ye L., Wang X., Song L., Wang Y.J., Liang H., Huang X.Y., Ho W.Z. (2008). Natural killer cells suppress full cycle HCV infection of human hepatocytes. J. Viral Hepat..

[44] Orr M.T., Lanier L.L. (2010). Natural killer cell education and tolerance. Cell.

[45] Carretero M., Palmieri G., Llano M., Tullio V., Santoni A., Geraghty D.E., Lopez-Botet M. (1998). Specific engagement of the CD94/NKG2-A killer inhibitory receptor by the HLA-E class Ib molecule induces SHP-1 phosphatase recruitment to tyrosine-phosphorylated NKG2-A: Evidence for receptor function in heterologous transfectants. Eur. J. Immunol..

[46] Moretta A., Moretta L. (1997). HLA class I specific inhibitory receptors. Curr. Opin. Immunol..

[47] Borrego F., Kabat J., Kim D.K., Lieto L., Maasho K., Pena J., Solana R., Coligan J.E. (2002). Structure and function of major histocompatibility complex (MHC) class I specific receptors expressed on human natural killer (NK) cells. Mol. Immunol..

[48] Kang W., Shin E.C. (2012). Interferon-induced MHC class I expression is attenuated by Hepatitis C Virus. Hepatology.

[49] Abel A.M., Yang C., Thakar M.S., Malarkannan S. (2018). Natural Killer Cells: Development, Maturation, and Clinical Utilization. Front. Immunol..

[50] Rolle A., Meyer M., Calderazzo S., Jager D., Momburg F. (2018). Distinct HLA-E Peptide Complexes Modify Antibody-Driven Effector Functions of Adaptive NK Cells. Cell Rep..

[51] Melsen J.E., Lugthart G., Lankester A.C., Schilham M.W. (2016). Human Circulating and Tissue-Resident CD56(bright) Natural Killer Cell Populations. Front. Immunol..

[52] Michel T., Poli A., Cuapio A., Briquemont B., Iserentant G., Ollert M., Zimmer J. (2016). Human CD56(bright) NK Cells: An Update. J. Immunol..

[53] Bjorkstrom N.K., Ljunggren H.G., Sandberg J.K. (2010). CD56 negative NK cells: Origin, function, and role in chronic viral disease. Trends Immunol..

[54] Milush J.M., Lopez-Verges S., York V.A., Deeks S.G., Martin J.N., Hecht F.M., Lanier L.L., Nixon D.F. (2013). CD56(neg)CD16(+) NK cells are activated mature NK cells with impaired effector function during HIV-1 infection. Retrovirology.

[55] Gonzalez V.D., Falconer K., Bjorkstrom N.K., Blom K.G., Weiland O., Ljunggren H.G., Alaeus A., Sandberg J.K. (2009). Expansion of Functionally Skewed CD56-Negative NK Cells in Chronic Hepatitis C Virus Infection: Correlation with Outcome of Pegylated IFN-alpha and Ribavirin Treatment. J. Immunol..

[56] Carrega P., Ferlazzo G. (2012). Natural killer cell distribution and trafficking in human tissues. Front. Immunol..

[57] Mikulak J., Bruni E., Oriolo F., Di Vito C., Mavilio D. (2019). Hepatic Natural Killer Cells: Organ-Specific Sentinels of Liver Immune Homeostasis and Physiopathology. Front. Immunol..

[58] Hudspeth K., Donadon M., Cimino M., Pontarini E., Tentorio P., Preti M., Hong M., Bertoletti A., Bicciato S., Invernizzi P. (2016). Human liver-resident CD56(bright)/CD16(neg) NK cells are retained within hepatic sinusoids via the engagement of CCR5 and CXCR6 pathways. J. Autoimmun..

[59] Cuff A.O., Robertson F.P., Stegmann K.A., Pallett L.J., Maini M.K., Davidson B.R., Male V. (2016). Eomeshi NK Cells in Human Liver Are Long-Lived and Do Not Recirculate but Can Be Replenished from the Circulation. J. Immunol..

[60] Yoon J.C., Yang C.M., Song Y., Lee J.M. (2016). Natural killer cells in hepatitis C: Current progress. World J. Gastroenterol..

[61] Marra F., Tacke F. (2014). Roles for Chemokines in Liver Disease. Gastroenterology.

[62] Saiman Y., Friedman S.L. (2012). The role of chemokines in acute liver injury. Front. Physiol..

[63] Wakita T., Pietschmann T., Kato T., Date T., Miyamoto M., Zhao Z., Murthy K., Habermann A., Krausslich H.G., Mizokami M. (2005). Production of infectious hepatitis C virus in tissue culture from a cloned viral genome. Nat. Med..

[64] Stegmann K.A., Bjorkstrom N.K., Ciesek S., Lunemann S., Jaroszewicz J., Wiegand J., Malinski P., Dustin L.B., Rice C.M., Manns M.P. (2012). Interferon alpha-Stimulated Natural Killer Cells From Patients With Acute Hepatitis C Virus (HCV) Infection Recognize HCV-Infected and Uninfected Hepatoma Cells via DNAX accessory molecule-1. J. Infect. Dis..

[65] Yoon J.C., Lim J.B., Park J.H., Lee J.M. (2011). Cell-to-Cell Contact with Hepatitis C Virus-Infected Cells Reduces Functional Capacity of Natural Killer Cells. J. Virol..

[66] Holder K.A., Stapleton S.N., Gallant M.E., Russell R.S., Grant M.D. (2013). Hepatitis C Virus-Infected Cells Downregulate NKp30 and Inhibit Ex Vivo NK Cell Functions. J. Immunol..

[67] Sene D., Levasseur F., Abel M., Camous X., Rosenbereg A.R., Marche P.N., Cacoub P., Caillat-Zucman S. (2010). Hepatitis C Virus (Hcv) Evades Nkg2d-Dependent NK Cell Responses through Ns5a-Mediated Imbalance of Inflammatory Cytokines. Hepatology.

[68] Yang C.M., Yoon J.C., Park J.H., Lee J.M. (2017). Hepatitis C virus impairs natural killer cell activity via viral serine protease NS3. PLoS ONE.

[69] Sene D., Levasseur F., Abel M., Lambert M., Camous X., Hernandez C., Pene V., Rosenberg A.R., Jouvin-Marche E., Marche P.N. (2010). Hepatitis C Virus (HCV) Evades NKG2D-Dependent NK Cell Responses through NS5A-Mediated Imbalance of Inflammatory Cytokines. PLoS Pathog..

[70] Tseng C.T.K., Klimpel G.R. (2002). Binding of the hepatitis C virus envelope protein E2 to CD81 inhibits natural killer cell functions. J. Exp. Med..

[71] Nattermann J., Nischalke H.D., Hofmeister V., Ahlenstiel G., Zimmermann H., Leifeld L., Weiss E.H., Sauerbruch T., Spengler U. (2005). The HLA-A2 restricted T cell epitope HCV core 35-44 stabilizes HLA-E expression and inhibits cytolysis mediated by natural killer cells. Am. J. Pathol..

[72] Schulte D., Vogel M., Langhans B., Kramer B., Korner C., Nischalke H.D., Steinberg V., Michalk M., Berg T., Rockstroh J.K. (2009). The HLA-E(R)/HLA-E(R) genotype affects the natural course of hepatitis C virus (HCV) infection and is associated with HLA-E-restricted recognition of an HCV-derived peptide by interferon-gamma-secreting human CD8(+) T cells. J. Infect. Dis..

[73] Irshad M., Khushboo I., Singh S., Singh S. (2008). Hepatitis C Virus (HCV): A Review of Immunological Aspects. Int. Rev. Immunol..

[74] Takahashi K., Asabe S., Wieland S., Garaigorta U., Gastaminza P., Isogawa M., Chisari F.V. (2010). Plasmacytoid dendritic cells sense hepatitis C virus-infected cells, produce interferon, and inhibit infection. Proc. Natl. Acad. Sci. USA.

[75] Jinushi M., Takehara T., Tatsumi T., Kanto T., Groh V., Spies T., Suzuki T., Miyagi T., Hayashi N. (2003). Autocrine/paracrine IL-15 that is required for type I IFN-mediated dendritic cell expression of MHC class I-related chain A and B is impaired in hepatitis C virus infection. J. Immunol..

[76] Lucas M., Schachterle W., Oberle K., Aichele P., Diefenbach A. (2007). Dendritic cells prime natural killer cells by trans-presenting interleukin 15. Immunity.

[77] Ma C.J., Ni L., Zhang Y., Zhang C.L., Wu X.Y., Atia A.N., Thayer P., Moorman J.P., Yao Z.Q. (2011). PD-1 negatively regulates interleukin-12 expression by limiting STAT-1 phosphorylation in monocytes/macrophages duringchronic hepatitis C virus infection. Immunology.

[78] Wang J.M., Shi L., Ma C.J., Ji X.J., Ying R.S., Wu X.Y., Wang K.S., Li G., Moorman J.P., Yao Z.Q. (2013). Differential regulation of interleukin-12 (IL-12)/IL-23 by Tim-3 drives T(H)17 cell development during hepatitis C virus infection. J. Virol..

[79] Zhang Y., Ma C.J., Wang J.M., Ji X.J., Wu X.Y., Jia Z.S., Moorman J.P., Yao Z.Q. (2011). Tim-3 negatively regulates IL-12 expression by monocytes in HCV infection. PLoS ONE.

[80] Khakoo S.I., Thio C.L., Martin M.P., Brooks C.R., Gao X., Astemborski J., Cheng J., Goedert J.J., Vlahov D., Hilgartner M. (2004). HLA and NK cell inhibitory receptor genes in resolving hepatitis C virus infection. Science.

[81] Romero V., Azocar J., Zuniga J., Clavijo O.P., Terreros D., Gu X., Husain Z., Chung R.T., Amos C., Yunis E.J. (2008). Interaction of NK inhibitory receptor genes with HLA-C and MHC class II alleles in Hepatitis C virus infection outcome. Mol. Immunol..

[82] Moesta A.K., Norman P.J., Yawata M., Yawata N., Gleimer M., Parham P. (2008). Synergistic polymorphism at two positions distal to the ligand-binding site makes KIR2DL2 a stronger receptor for HLA-C than KIR2DL3. J. Immunol..

[83] Gardiner C.M. (2015). NK cell function and receptor diversity in the context of HCV infection. Front. Microbiol..

[84] Dring M.M., Morrison M.H., McSharry B.P., Guinan K.J., Hagan R., Irish H.C.V.R.C., O’Farrelly C., Gardiner C.M. (2011). Innate immune genes synergize to predict increased risk of chronic disease in hepatitis C virus infection. Proc. Natl. Acad. Sci. USA.

[85] Strong R.K., Holmes M.A., Morris D.L., Braun L., Lee N., Geraghty D.E. (2002). HLA-E allelic variants: Correlating differential expression, peptide affinities, crystal structures and thermal stabilities. Tissue Antigens.

[86] Amadei B., Urbani S., Cazaly A., Fisicaro P., Zerbini A., Ahmed P., Missale G., Ferrari C., Khakoo S.I. (2010). Activation of natural killer cells during acute infection with hepatitis C virus. Gastroenterology.

[87] Alter G., Jost S., Rihn S., Reyor L.L., Nolan B.E., Ghebremichael M., Bosch R., Altfeld M., Lauer G.M. (2011). Reduced frequencies of NKp30+NKp46+, CD161+, and NKG2D+NK cells in acute HCV infection may predict viral clearance. J. Hepatol..

[88] Kokordelis P., Kramer B., Korner C., Boesecke C., Voigt E., Ingiliz P., Glassner A., Eisenhardt M., Wolter F., Kaczmarek D. (2014). An Effective Interferon-Gamma-Mediated Inhibition of Hepatitis C Virus Replication by Natural Killer Cells Is Associated With Spontaneous Clearance of Acute Hepatitis C in Human Immunodeficiency Virus-Positive Patients. Hepatology.

[89] Golden-Mason L., Madrigal-Estebas L., McGrath E., Conroy M.J., Ryan E.J., Hegarty J.E., O’Farrelly C., Doherty D.G. (2008). Altered natural killer cell subset distributions in resolved and persistent hepatitis C virus infection following single source exposure. Gut.

[90] Dessouki O., Kamiya Y., Nagahama H., Tanaka M., Suzu S., Sasaki Y., Okada S. (2010). Chronic hepatitis C viral infection reduces NK cell frequency and suppresses cytokine secretion: Reversion by anti-viral treatment. Biochem. Biophys. Res. Commun..

[91] Ahlenstiel G., Titerence R.H., Koh C., Edlich B., Feld J.J., Rotman Y., Ghany M.G., Hoofnagle J.H., Liang T.J., Heller T. (2010). Natural Killer Cells Are Polarized Toward Cytotoxicity in Chronic Hepatitis C in an Interferon-Alfa-Dependent Manner. Gastroenterology.

[92] Oliviero B., Varchetta S., Paudice E., Michelone G., Zaramella M., Mavilio D., De Filippi F., Bruno S., Mondelli M.U. (2009). Natural killer cell functional dichotomy in chronic hepatitis B and chronic hepatitis C virus infections. Gastroenterology.

[93] Miyagi T., Gil M.P., Wang X., Louten J., Chu W.M., Biron C.A. (2007). High basal STAT4 balanced by STAT1 induction to control type 1 interferon effects in natural killer cells. J. Exp. Med..

[94] Miyagi T., Takehara T., Nishio K., Shimizu S., Kohga K., Li W., Tatsumi T., Hiramatsu N., Kanto T., Hayashi N. (2010). Altered interferon-alpha-signaling in natural killer cells from patients with chronic hepatitis C virus infection. J. Hepatol..

[95] Edlich B., Ahlenstiel G., Azpiroz A.Z., Stoltzfus J., Noureddin M., Serti E., Feld J.J., Liang T.J., Rotman Y., Rehermann B. (2012). Early changes in interferon signaling define natural killer cell response and refractoriness to interferon-based therapy of hepatitis C patients. Hepatology.

[96] Pembroke T., Christian A., Jones E., Hills R.K., Wang E.C., Gallimore A.M., Godkin A. (2014). The paradox of NKp46+ natural killer cells: Drivers of severe hepatitis C virus-induced pathology but in-vivo resistance to interferon alpha treatment. Gut.

[97] Kramer B., Korner C., Kebschull M., Glassner A., Eisenhardt M., Nischalke H.D., Alexander M., Sauerbruch T., Spengler U., Nattermann J. (2012). Natural killer p46High expression defines a natural killer cell subset that is potentially involved in control of hepatitis C virus replication and modulation of liver fibrosis. Hepatology.

[98] Jinushi M., Takehara T., Tatsumi T., Kanto T., Miyagi T., Suzuki T., Kanazawa Y., Hiramatsu N., Hayashi N. (2004). Negative regulation of NK cell activities by inhibitory receptor CD94/NKG2A leads to altered NK cell-induced modulation of dendritic cell functions in chronic hepatitis C virus infection. J. Immunol..

[99] Szereday L., Meggyes M., Halasz M., Szekeres-Bartho J., Par A., Par G. (2016). Immunological changes in different patient populations with chronic hepatitis C virus infection. World J. Gastroenterol..

[100] Bozzano F., Picciotto A., Costa P., Marras F., Fazio V., Hirsch I., Olive D., Moretta L., De Maria A. (2011). Activating NK cell receptor expression/function (NKp30, NKp46, DNAM-1) during chronic viraemic HCV infection is associated with the outcome of combined treatment. Eur. J. Immunol..

[101] Beziat V., Dalgard O., Asselah T., Halfon P., Bedossa P., Boudifa A., Hervier B., Theodorou I., Martinot M., Debre P. (2012). CMV drives clonal expansion of NKG2C(+) NK cells expressing self-specific KIRs in chronic hepatitis patients. Eur. J. Immunol..

[102] Zhang C., Wang X.M., Li S.R., Twelkmeyer T., Wang W.H., Zhang S.Y., Wang S.F., Chen J.Z., Jin X., Wu Y.Z. (2019). NKG2A is a NK cell exhaustion checkpoint for HCV persistence. Nat. Commun..

[103] Cosgrove C., Berger C.T., Kroy D.C., Cheney P.C., Ghebremichael M., Aneja J., Tomlinson M., Kim A.Y., Lauer G.M., Alter G. (2014). Chronic HCV Infection Affects the NK Cell Phenotype in the Blood More than in the Liver. PLoS ONE.

[104] Rivero-Juarez A., Gonzalez R., Camacho A., Manzanares-Martin B., Caruz A., Martinez-Peinado A., Torre-Cisneros J., Pineda J.A., Pena J., Rivero A. (2013). Natural Killer KIR3DS1 Is Closely Associated with HCV Viral Clearance and Sustained Virological Response in HIV/HCV Patients. PLoS ONE.

[105] Lopez-Vazquez A., Rodrigo L., Martinez-Borra J., Perez R., Rodriguez M., Fdez-Morera J.L., Fuentes D., Rodriguez-Rodero S., Gonzalez S., Lopez-Larrea C. (2005). Protective effect of the HLA-Bw4I80 epitope and the killer cell immunoglobulin-like receptor 3DS1 gene against the development of hepatocellular carcinoma in patients with hepatitis C virus infection. J. Infect. Dis..

[106] Lunemann S., Schobel A., Kah J., Fittje P., Holzemer A., Langeneckert A.E., Hess L.U., Poch T., Martrus G., Garcia-Beltran W.F. (2018). Interactions Between KIR3DS1 and HLA-F Activate Natural Killer Cells to Control HCV Replication in Cell Culture. Gastroenterology.

[107] De Groen R.A., Groothuismink Z.M.A., van Oord G., Kootstra N.A., Janssen H.L.A., Prins M., Schinkel J., Boonstra A. (2017). NK cells in self-limited HCV infection exhibit a more extensively differentiated, but not memory-like, repertoire. J. Viral Hepat..

[108] Krueger P.D., Narayanan S., Surette F.A., Brown M.G., Sung S.J., Hahn Y.S. (2017). Murine liver-resident group 1 innate lymphoid cells regulate optimal priming of anti-viral CD8+ T cells. J. Leukoc. Biol..

[109] Peng H., Tian Z. (2017). Natural Killer Cell Memory: Progress and Implications. Front. Immunol..

[110] Bjorkstrom N.K., Lindgren T., Stoltz M., Fauriat C., Braun M., Evander M., Michaelsson J., Malmberg K.J., Klingstrom J., Ahlm C. (2011). Rapid expansion and long-term persistence of elevated NK cell numbers in humans infected with hantavirus. J. Exp. Med..

[111] Reeves R.K., Li H., Jost S., Blass E., Li H., Schafer J.L., Varner V., Manickam C., Eslamizar L., Altfeld M. (2015). Antigen-specific NK cell memory in rhesus macaques. Nat. Immunol..

[112] Hammer Q., Ruckert T., Borst E.M., Dunst J., Haubner A., Durek P., Heinrich F., Gasparoni G., Babic M., Tomic A. (2018). Peptide-specific recognition of human cytomegalovirus strains controls adaptive natural killer cells. Nat. Immunol..

[113] Foley B., Cooley S., Verneris M.R., Curtsinger J., Luo X., Waller E.K., Anasetti C., Weisdorf D., Miller J.S. (2012). Human cytomegalovirus (CMV)-induced memory-like NKG2C(+) NK cells are transplantable and expand in vivo in response to recipient CMV antigen. J. Immunol..

[114] Paust S., Gill H.S., Wang B.Z., Flynn M.P., Moseman E.A., Senman B., Szczepanik M., Telenti A., Askenase P.W., Compans R.W. (2010). Critical role for the chemokine receptor CXCR6 in NK cell-mediated antigen-specific memory of haptens and viruses. Nat. Immunol..

[115] Abdul-Careem M.F., Lee A.J., Pek E.A., Gill N., Gillgrass A.E., Chew M.V., Reid S., Ashkar A.A. (2012). Genital HSV-2 infection induces short-term NK cell memory. PLoS ONE.

[116] Wijaya R.S., Read S.A., Truong N.R., Han S., Chen D., Shahidipour H., Fewings N.L., Schibeci S., Azardaryany M.K., Parnell G.P. (2020). HBV vaccination and HBV infection induces HBV-specific natural killer cell memory. Gut.

[117] Manns M.P., McHutchison J.G., Gordon S.C., Rustgi V.K., Shiffman M., Reindollar R., Goodman Z.D., Koury K., Ling M.H., Albrecht J.K. (2001). Peginterferon alfa-2b plus ribavirin compared with interferon alfa-2b plus ribavirin for initial treatment of chronic hepatitis C: A randomised trial. Lancet.

[118] Poordad F., McCone J., Bacon B.R., Bruno S., Manns M.P., Sulkowski M.S., Jacobson I.M., Reddy K.R., Goodman Z.D., Boparai N. (2011). Boceprevir for untreated chronic HCV genotype 1 infection. N. Engl. J. Med..

[119] Jacobson I.M., McHutchison J.G., Dusheiko G., Di Bisceglie A.M., Reddy K.R., Bzowej N.H., Marcellin P., Muir A.J., Ferenci P., Flisiak R. (2011). Telaprevir for previously untreated chronic hepatitis C virus infection. N. Engl. J. Med..

[120] Bourliere M., Gordon S.C., Flamm S.L., Cooper C.L., Ramji A., Tong M., Ravendhran N., Vierling J.M., Tran T.T., Pianko S. (2017). Sofosbuvir, Velpatasvir, and Voxilaprevir for Previously Treated HCV Infection. N. Engl. J. Med..

[121] Zeuzem S., Foster G.R., Wang S., Asatryan A., Gane E., Feld J.J., Asselah T., Bourliere M., Ruane P.J., Wedemeyer H. (2018). Glecaprevir-Pibrentasvir for 8 or 12 Weeks in HCV Genotype 1 or 3 Infection. N. Engl. J. Med..

[122] Ascierto M.L., Bozzano F., Bedognetti D., Marras F., Schechterly C., Matsuura K., Picciotto A., Marenco S., Zhao Y.D., DeGiorgi V. (2015). Inherent transcriptional signatures of NK cells are associated with response to IFN alpha plus rivabirin therapy in patients with Hepatitis C Virus. J. Transl. Med..

[123] Hu X.L., Jiang Y.F., Li X.R., Gao Y.H., Guo X.L., Chi X.M., Yan H.Q., Feng J.Y., Zhong J., Sun B. (2014). Long-Term Effect on Natural Killer Cells by Interferon-alpha Therapy on the Outcomes of HCV Infection. J. Interferon Cytokine Res..

[124] Sarasin-Filipowicz M., Oakeley E.J., Duong F.H., Christen V., Terracciano L., Filipowicz W., Heim M.H. (2008). Interferon signaling and treatment outcome in chronic hepatitis C. Proc. Natl. Acad. Sci. USA.

[125] Conry S.J., Meng Q.L., Hardy G., Yonkers N.L., Sugalski J.M., Hirsch A., Davitkov P., Compan A., Falck-Ytter Y., Blanton R.E. (2012). Genetically Associated CD16(+)56(-) Natural Killer Cell Interferon (IFN)-alpha R Expression Regulates Signaling and Is Implicated in IFN-alpha-Induced Hepatitis C Virus Decline. J. Infect. Dis..

[126] Vidal-Castineira J.R., Lopez-Vazquez A., Diaz-Pena R., Alonso-Arias R., Martinez-Borra J., Perez R., Fernandez-Suarez J., Melon S., Prieto J., Rodrigo L. (2010). Effect of killer immunoglobulin-like receptors in the response to combined treatment in patients with chronic hepatitis C virus infection. J. Virol..

[127] Suppiah V., Gaudieri S., Armstrong N.J., O’Connor K.S., Berg T., Weltman M., Abate M.L., Spengler U., Bassendine M., Dore G.J. (2011). IL28B, HLA-C, and KIR variants additively predict response to therapy in chronic hepatitis C virus infection in a European Cohort: A cross-sectional study. PLoS Med..

[128] Oliviero B., Mele D., Degasperi E., Aghemo A., Cremonesi E., Rumi M.G., Tinelli C., Varchetta S., Mantovani S., Colombo M. (2013). Natural killer cell dynamic profile is associated with treatment outcome in patients with chronic HCV infection. J. Hepatol..

[129] Werner J.M., Serti E., Chepa-Lotrea X., Stoltzfus J., Ahlenstiel G., Noureddin M., Feld J.J., Liang T.J., Rotman Y., Rehermann B. (2014). Ribavirin improves the IFN-γ response of natural killer cells to IFN-based therapy of hepatitis C virus infection. Hepatology.

[130] Falade-Nwulia O., Suarez-Cuervo C., Nelson D.R., Fried M.W., Segal J.B., Sulkowski M.S. (2017). Oral Direct-Acting Agent Therapy for Hepatitis C Virus Infection: A Systematic Review. Ann. Intern. Med..

[131] Martin B., Hennecke N., Lohmann V., Kayser A., Neumann-Haefelin C., Kukolj G., Böcher W.O., Thimme R. (2014). Restoration of HCV-specific CD8+ T cell function by interferon-free therapy. J. Hepatol..

[132] Serti E., Chepa-Lotrea X., Kim Y.J., Keane M., Fryzek N., Liang T.J., Ghany M., Rehermann B. (2015). Successful Interferon-Free Therapy of Chronic Hepatitis C Virus Infection Normalizes Natural Killer Cell Function. Gastroenterology.

[133] Spaan M., van Oord G., Kreefft K., Hou J., Hansen B.E., Janssen H.L., de Knegt R.J., Boonstra A. (2016). Immunological Analysis During Interferon-Free Therapy for Chronic Hepatitis C Virus Infection Reveals Modulation of the Natural Killer Cell Compartment. J. Infect. Dis..

[134] Golden-Mason L., McMahan R.H., Kriss M.S., Kilgore A.L., Cheng L., Dran R.J., Wieland A., Rosen H.R. (2018). Early and late changes in natural killer cells in response to ledipasvir/sofosbuvir treatment. Hepatol. Commun..

[135] Alao H., Cam M., Keembiyehetty C., Zhang F., Serti E., Suarez D., Park H., Fourie N.H., Wright E.C., Henderson W.A. (2018). Baseline Intrahepatic and Peripheral Innate Immunity are Associated with Hepatitis C Virus Clearance During Direct-Acting Antiviral Therapy. Hepatology.

[136] Strunz B., Hengst J., Deterding K., Manns M.P., Cornberg M., Ljunggren H.G., Wedemeyer H., Björkström N.K. (2018). Chronic hepatitis C virus infection irreversibly impacts human natural killer cell repertoire diversity. Nat. Commun..

[137] Santangelo L., Bordoni V., Montaldo C., Cimini E., Zingoni A., Battistelli C., D’Offizi G., Capobianchi M.R., Santoni A., Tripodi M. (2018). Hepatitis C virus direct-acting antivirals therapy impacts on extracellular vesicles microRNAs content and on their immunomodulating properties. Liver Int..

[138] Chu P.S., Nakamoto N., Taniki N., Ojiro K., Amiya T., Makita Y., Murata H., Yamaguchi A., Shiba S., Miyake R. (2017). On-treatment decrease of NKG2D correlates to early emergence of clinically evident hepatocellular carcinoma after interferon-free therapy for chronic hepatitis C. PLoS ONE.

